# The complete mitochondrial genome of *Aconitum kusnezoffii* Rchb. (Ranales, Ranunculaceae)

**DOI:** 10.1080/23802359.2021.1882894

**Published:** 2021-03-11

**Authors:** Sheng-Nan Li, Yan-Yun Yang, Liang Xu, Yan-Ping Xing, Rong Zhao, Wu-Liji Ao, Ting-Ting Zhang, Da-Chuan Zhang, Yue-Yue Song, Gui-Hua Bao, Ting-Guo Kang

**Affiliations:** a School of Pharmacy, Liaoning University of Traditional Chinese Medicine, Dalian, China; b School of Mongol Medicine, Inner Mongolia University for Nationalities, Tongliao, China

**Keywords:** Mitochondrial genome, *Aconitum kusnezoffii*, Ranunculaceae

## Abstract

*Aconitum kusnezoffii* Rchb. is a medicinal plant in the Ranunculaceae family. In this study, we report the first complete mitochondrial genome of *A. kusnezoffii*. The total length of the mitochondrial genome of *A. kusnezoffii* is 440,720 bp and the GC content of 46.85%. The mitochondrial genome contained 37 protein-coding genes, 29 tRNAs, and three rRNAs. These data will provide the basis for the systematic evolutionary analysis of Ranunculaceae.

There are more than 2000 species of Ranunculaceae recorded in the world (Cossard et al. 2016). They are distributed on all continents of the world except Antarctica, especially in temperate, cold temperate, and alpine regions. Ranunculaceae plants contain a variety of chemical components, many of which can be used as medicinal plants. *Aconitum kusnezoffii* Rchb. is a poisonous medicinal plant and has a long history of clinical application. At present, the research on *A. kusnezoffii* mainly focuses on chemical composition (Zan et al. 2018), efficacy–toxicity relation (Zhang et al. 2018), and safety dose research (Kim et al. 2012). However, the genetics and molecular biology of *A. kusnezoffii* are poorly understood, which has hindered research on the phylogenetic and molecular mechanism research.

Genomic DNA was obtained from fresh leaves of *A. kusnezoffii* collected from Dalian, China (E 121°87′63.24″, N 39°06′18.72″) using the Illumina TruSeq™ Nano DNA Sample Prep Kit method. The voucher specimen (*A. kusnezoffii* number: 10162200520005LY) and genomic DNA were deposited at the herbarium of Liaoning University of Traditional Chinese Medicine. Mitochondrial genome sequencing was performed using Illumina NovaSeq platform (Illumina, San Diego, CA) and the Nanopore platform (Oxford Nanopore Technologies, Oxford Park, UK). First, ABySS v2.0.2 (Simpson et al. 2009) was used for genome assembly of multiple-Kmer parameters, and an optimal assembly result was obtained. Second, BLASR (Chaisson and Tesler 2012) was used to map the preliminary assembly results to the Nanopore long reads. Then, SPAdes v3.10.1 (Bankevich et al. 2012) was used to assemble them together to construct contigs (scaffolds). Finally, all aligned Nanopore reads were extracted to perform self-correction and mt genome de novo assembly using the Canu v2.0 (Koren et al. 2017) package, followed by error correction using the Pilon v1.21. The Nanopore assembled sequences were then checked if the sequences have overlap and connection between them.

The paired-end (PE) libraries with an insert size of 450 bp were subjected to the Illumina platform and Nanopore platform, which generated 24.9 million reads. The length of PE was 150 bp × 2. The assembled mitochondrial genome was a closed circular molecule with the length of 440,720 bp and the GC content of 46.85%. The mitochondrial genome contained 37 protein-coding genes, 29 tRNAs, and three rRNAs (*rrn18*, *rrn26*, *rrn5*). The total length of the protein-coding genes was 31,836 bp, accounting for 7.22% of the total length of the mitochondrial genome. The average length of tRNAs was 73 bp, while the average length of rRNAs was 2120 bp. In addition, we found seven genes (*nad1*, *nad7*, *nad4*, *ccmFC*, *nad2*, *nad5*, *rps3*) containing 16 introns in total.

The MUMmer (Marcais et al. 2018) and BLAT software (Kent 2002) were used to do global alignment and local alignment between sample sequence and the reference genome under default parameters, and then manually optimized. The maximum-likelihood (ML) methods were performed for the genome-wide phylogenetic analyses using PhyML 3.0, respectively. Nucleotide substitution model selection was estimated with jModelTest 2.1.10 and Smart Model Selection in PhyML 3.0. The model GTR + I+G was selected for the ML analyses with 1000 bootstrap (BS) replicates to calculate the BS values. The results tree was treated with iTOL 3.4.3. We modeled the ML tree with 23 other species (Figure 1). It was found that *A. kusnezoffii* and *Anemone maxima* (Park and Park 2020) from the order Ranunculales clustered closely together into one branch, which had 100% BS values. These data provide references for further study of the evolutionary history of *A. kusnezoffii.*

**Figure 1.**
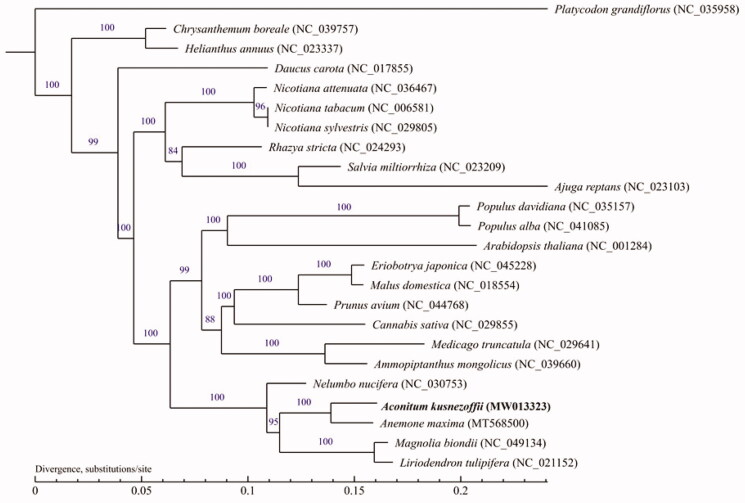
Maximum-likelihood (ML) tree based on the mitogenome sequence of *Aconitum kusnezoffii* with 23 other species, the bootstrap supports are shown on each node.

## Data Availability

The genome sequence data that support the findings of this study are openly available in GenBank of NCBI at (https://www.ncbi.nlm.nih.gov/) under the accession no. MW013323. The associated BioProject, SRA, and Bio-Sample numbers are PRJNA679444, SRX9535583, and SRS7742053, respectively.
